# Peptide modified manganese-doped iron oxide nanoparticles as a sensitive fluorescence nanosensor for non-invasive detection of trypsin activity *in vitro* and *in vivo*†

**DOI:** 10.1039/d0ra08171j

**Published:** 2021-01-11

**Authors:** Yu Fu, Lin Liu, Xiaodong Li, Hongda Chen, Zhenxin Wang, Wensheng Yang, Hua Zhang, Huimao Zhang

**Affiliations:** College of Chemistry, Jilin University Changchun 130021 P. R. China; Department of Radiology, The First Hospital of Jilin University Changchun 130021 P. R. China huimao@jlu.edu.cn; State Key Laboratory of Electroanalytical Chemistry, Changchun Institute of Applied Chemistry, Chinese Academy of Sciences Changchun 130022 P. R. China chenhongda@ciac.ac.cn zhanghua@ciac.ac.cn +86-431-85262243 +86-431-85262757

## Abstract

Herein, a fluorescence turn-on nanosensor (MnIO@pep-FITC) has been proposed for detecting trypsin activity *in vitro* and *in vivo* through covalently immobilizing an FITC modified peptide substrate of trypsin (pep-FITC) on manganese-doped iron oxide nanoparticle (MnIO NP) surfaces *via* a polyethylene glycol (PEG) crosslinker. The conjugation of pep-FITC with MnIO NPs results in the quenching of FITC fluorescence. After trypsin cleavage, the FITC moiety is released from the MnIO NP surface, leading to a remarkable recovery of FITC fluorescence signal. Under the optimum experimental conditions, the recovery ratio of FITC fluorescence intensity is linearly dependent on the trypsin concentration in the range of 2 to 100 ng mL^−1^ in buffer and intracellular trypsin in the lysate of 5 × 10^2^ to 1 × 10^4^ HCT116 cells per mL, respectively. The detection limit of trypsin is 0.6 ng mL^−1^ in buffer or 359 cells per mL HCT116 cell lysate. The MnIO@pep-FITC is successfully employed to noninvasively monitor trypsin activity in the ultrasmall (*ca.* 4.9 mm^3^ in volume) BALB/c nude mouse-bearing HCT116 tumor by *in vivo* fluorescence imaging with external magnetic field assistance, demonstrating that it has excellent practicability.

## Abstract 2

A fluorescence nanosensor has been proposed for detecting trypsin activity through the peptide substrate of trypsin on manganese-doped iron oxide nanoparticles.

## Graphical abstract

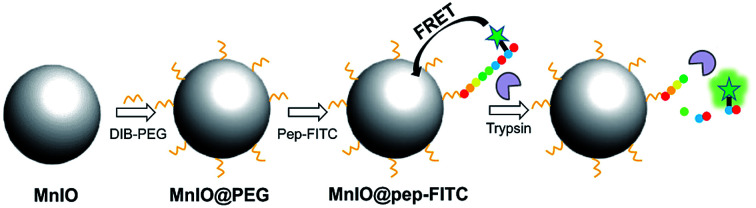

## 1. Introduction

As paradigms of enzyme catalysis, serine (Ser) proteases play critical roles in a variety of biological and physiological processes such as cell differentiation, cell apoptosis, blood coagulation, atherosclerosis, inflammation and cancer.^1–6^ The Ser proteases normally contain a nucleophilic Ser residue at their active site, which has nucleophilic activity against the peptide bond resulting in the digestion of some proteins implicated in vital activities. Trypsin (EC 3.4.21.4), a kind of Ser protease produced by the pancreas, is the most popular digestive enzyme, which is not only involved in the digestion of dietary proteins but also induces proteolytic cascades by activating other proteases, such as matrix metalloproteinases (MMPs) through the selective hydrolysis of polypeptide chains of arginine (Arg) or lysine (Lys) in the C-terminal.^7–9^ It is found that trypsin expression is significantly increased in several human cancer cells of the stomach, colon, lung and breast.^10^ Recently, trypsin has been regarded as a potential prognosticator for cancer patients because dysfunction of trypsin has been linked to the malignancy of several tumors including colorectal cancer (CRC), gastric cancer and pancreatic cancer.^9,11–16^ For instance, 5 year survival rate of patients with trypsin-positive CRC is lower than that of patients with trypsin-negative CRC.^11^ Therefore, timely detection of trypsin activity is critical for diagnosis and treatment of CRC.

At present, a large number of methods/assays have been developed for quantification of trypsin levels *in vitro* including chromatography,^17^ radioimmunoassay^18^ and biosensors with different detection principles.^19–38^ Among of these techniques, peptide substrate-based biosensors have been considered as a promising way to determine trypsin activity in different matrixes since peptide substrates exhibit several unique merits, such as easy synthesis, low cost, high stability in harsh environments and suitable to conjugate/modify with other label molecules.^33–38^ For example, a variety of peptide-based fluorescence turn-on/off strategies have been developed for sensing trypsin activity with high sensitivity and selectivity.^32–34^ Unfortunately, most of as-developed sensing platforms are only capable of trypsin activity detection *in vitro*, which does not necessarily reflect the quantitative information on trypsin expression in a solid tumor. It is still a challenge to develop peptide-based biosensors for measurement of tumor-related trypsin activity *in vivo* since it is difficult to efficiently transport peptide substrate to tumor site.

Recently, magnetic nanoparticles (MNPs) have been successfully employed to fabricate Förster resonance energy transfer (FRET) sensing platforms for detecting MMP-9 activity both *in vitro* and *in vivo* because there is inherent spectral overlap interference of fluorescent dyes and MNPs.^39^ The MNP-based FRET sensing platform enables to quantitatively map MMP-9 activity across the entire tumor because MNPs can efficiently accumulate in tumors through the enhanced permeability and retention (EPR) effect. In particular, the accumulation amount of MNPs in the tumor site can be further enhanced with the aid of an external magnetic field (MF).^40–42^ The phenomenon may increase the detection sensitivity of MNP-based FRET sensing platforms.

Herein, a peptide-functionalized manganese-doped iron oxide nanoparticle-based fluorescence turn-on sensing platform (MnIO@pep-FITC) has been constructed for noninvasive detection of trypsin activity *in vivo* through conjugation of FITC modified peptide substrate on the MnIO NP surface. Under the specific digestion of trypsin, the FITC was liberated from MnIO@pep-FITC, and quenched fluorescence could be recovered in response to trypsin activity. With the aid of an external MF, we demonstrate that MnIO@pep-FITC can be used to visualize ultrasmall (*ca.* 4.9 mm^3^ in volume) trypsin-positive subcutaneous tumor (HCT116) in BALB/c nude mouse through intravenous administration, showing great promising application in the diagnosis of tumor.

## 2. Experimental section

### 2.1 Materials and reagents

Sodium oleate was purchased from TCI Development Co. (Shanghai, China). Oleic acid (OA, 90 wt%) and 1-octadecene (ODE, 90 wt%) were purchased from Sigma-Aldrich Co. (St. Louis, USA). 3,4-dihydroxy benzyl amine-PEG-COOH (DIB-PEG-COOH, MW = 2000) was synthesized by Shanghai Ponsure Biotech. Ltd. (Shanghai, China). Fluorescein isothiocyanate modified peptide, GHCLRKGK(5-FITC)G (pep-FITC) was purchased from ChinaPeptides Ltd (Shanghai, China). Fetal bovine serum (FBS) was purchased from Gibco Ltd. (New York, USA). 3-(4,5-Dimethyl-2-thiazolyl)-2,5-diphenyl-2*H*-tetrazolium bromide (MTT) and RPMI 1640 culture medium were purchased from Beijing Dingguo Biotechnology Ltd. (Beijing, China). McCoy's 5A culture medium was purchased from KeyGen Biotech. Ltd. (Jiangsu, China). The HCT116 and NCM460 cells were purchased from Shanghai Cell Bank, Chinese Academy of Sciences (Shanghai, China). BALB/c nude mice (female, 6–8 weeks old) with average body weight of 18 g were purchased from Vital River Ltd. (Beijing, China). Other reagents (analytical grade) were purchased from Beijing Chemical Reagents Co. (Beijing, China). Milli-Q H_2_O (18.2 MΩ cm) was used in all experiments.

### 2.2 Fabrication of MnIO@pep-FITC

The OA capped MnFe_2_O_4_ nanoparticles (MnIO NPs) were firstly synthesized by the literature reported strategy with slight modifications (see ESI† for details).^43^ For transferring the hydrophilic OA capped MnIO NPs to aqueous medium, 20 mg OA capped MnIO NPs were mixed with 20 mg DIB-PEG-COOH in 5 mL CHCl_3_ and stirred at room temperature for 4 h. Then, the DIB-PEG-COOH modified MnIO NPs (MnIO@PEG) were collected by centrifugation (6000 rpm, 10 min), washed with the 10 mL mixture of hexane and CHCl_3_ (6000 rpm, 10 min, 3 times), and redispersed in 1 mL H_2_O.

For the preparation of MnIO@pep-FITC, the MnIO@PEG (1 mg mL^−1^) were mixed with 5 mmol L^−1^ sulfo-NHS (hydroxy-2,5-dioxopyrrolidine-3-sulfonicacid sodium salt) and 2 mmol L^−1^ EDC (1-ethyl-3-(3-dimethylaminopropyl)-carbodiimide) in 10 mL MES (2-morpholinoethanesulfonic acid) buffer (pH = 7.4, 10 mmol L^−1^). After incubated for 2 h, the activated MnIO@PEG were collected by centrifugation (6000 rpm, 10 min), and reacted with pep-FITC (0.1 mg mL^−1^ in 10 mL PBS (10 mmol L^−1^, pH 7.4)) under stirring for another 24 h. Finally, the pep-FITC conjugated MnIO@PEG (MnIO@pep-FITC) were purified by centrifugation at 4 °C (6000 rpm, 10 mL PBS, 3 times) and resuspended in 1 mL PBS.

### 2.3 Sensing performance of MnIO@pep-FITC in buffer

For trypsin detection, 100 μg mL^−1^ MnIO@pep-FITC were incubated with various concentrations of trypsin in 500 μL PBS (10 mmol L^−1^, pH 7.4) at 37 °C for 90 min. Subsequently, the fluorescence spectra of mixtures were measured on a QE65 Pro fiber optic spectrometer with 490 nm as the excitation wavelength. To investigate its selectivity, MnIO@pep-FITC were incubated with 100 ng mL^−1^ a series of enzymes including matrix metalloproteinases (MMP-2, MMP-7 and MMP-9), caspase-3 and caspase-9 under optimized conditions.

### 2.4 Intracellular trypsin activity detection

The HCT116 and NCM460 cells (1.5 × 10^4^ cells per well) were seeded in the 48-well microtiter plate and cultured in 300 μL fresh culture medium for 24 h. After washed with PBS (3 times), the cells were incubated with 100 μg mL^−1^ MnIO@pep-FITC in 300 μL fresh culture medium at 37 °C for various time (0, 1, 2, 3 and 4 h), respectively. Then, the fluorescence imaging was performed with a reconstructive Nikon Ti–S fluorescent microscope at the FITC channel. For quantitative measurements, the MnIO@pep-FITC stained cells were washed with PBS (300 μL, 3 times), detached from the 48-well plate, collected by centrifugation (1000 rpm, 5 min), redispersed in 100 μL lysis buffer, diluted by 400 μL PBS, and subjected to fluorescence measurement. In addition, to investigate the intracellular trypsin detection sensitivity, the unstained HCT116 cells were also treated as previously described, and then the cell lysates containing different cell numbers were incubated with 100 μg mL^−1^ MnIO@pep-FITC in 500 μL PBS at 37 °C for 90 min. The fluorescence spectra of mixture were then measured at the excitation wavelength of 490 nm. For the *in vitro* MF-assisted fluorescence imaging, a magnet was placed under the center of the cell culture dish while 100 μg mL^−1^ MnIO@pep-FITC were added into the dish. After co-cultured for 4 h, the cells were washed with 1 mL PBS (3 times), and subjected to image by a reconstructive Nikon Ti–S fluorescent microscope at the FITC channel.

### 2.5 *In vivo* toxicity evaluation

All animal procedures were performed in accordance with the Guidelines for Care and Use of Laboratory Animals of Jilin University and experiments were approved by the Animal Ethics Committee of Jilin University. For histology analysis, 200 μL 0.9 wt% NaCl solution with or without 10 mg kg^−1^ MnIO@pep-FITC were injected intravenously into the healthy mice as experimental group and control group, respectively. The healthy mice were anatomized at 30 days post-injection, and the major organs including heart, liver, spleen, lung and kidneys were collected for hematoxylin and eosin (H&E) staining. Furthermore, blood biochemistry assay was also employed to evaluate the biosafety of MnIO@pep-FITC.

### 2.6 *In vivo* fluorescence and magnetic resonance (MR) imaging

Female BALB/c nude mice (6 to 8 weeks old) were selected for establishing a HCT116 tumor model and injected subcutaneously with HCT116 cells (3 × 10^6^ cells in 100 μL PBS) on the right flanks of mice. A 0.5 T magnet was placed around the tumor site for generate external MF. For *in vivo* MR imaging, the HCT116 tumor-bearing mice (tumor size: 113 mm^3^) were injected intravenously with NaCl solution (0.9 wt%, 200 μL) containing 10 mg kg^−1^ MnIO@pep-FITC. After placed in the MF for 15 min, T_2_-weighted MR images of mice were collected by using a GE Signa 3 T MR unit with the following imaging parameters: repetition time (TR), 240 ms; echo time (TE), 15.9 ms; field of view, 120 mm × 72 mm; slice thickness, 2.0 mm. For *in vivo* fluorescence imaging, the tumor-bearing mice (tumor size: 4.9 mm and 126 mm^3^) were injected intravenously with NaCl solution (0.9 wt%, 200 μL) containing 10 mg kg^−1^ MnIO@pep-FITC. Subsequently, the mice were placed in the MF for 15 min and then the *in vivo* fluorescence imaging was collected by the Davinch Invivo HR imaging system at predetermined time intervals (excitation wavelength: 490 nm; emission wavelength: 520 nm, exposure time: 5 s). For quantitative analysis, the regions of interest (ROI) were drawn over tumors and measured by the ImageJ software. The tumor volumes were calculated according to the following formula: tumor volume (*V*) = (length × width × hight × π)/6.

## 3. Results and discussion

### 3.1 Synthesis and characterization of MnIO@pep-FITC

**Scheme 1:**
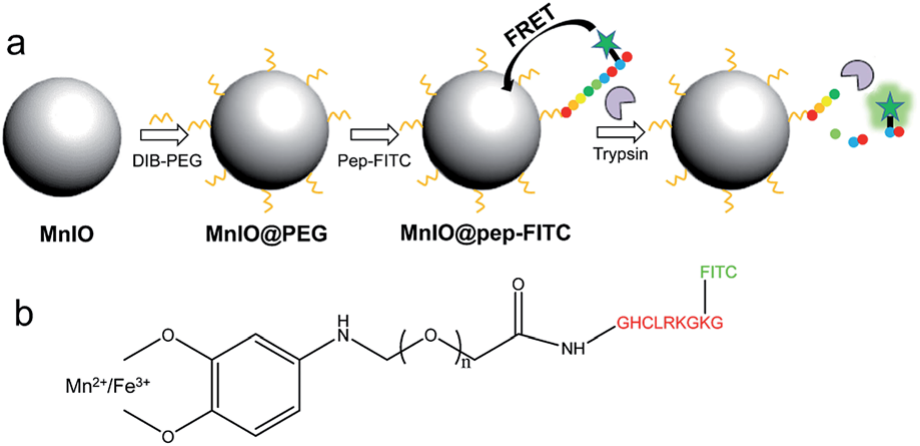
(a) The construction procedure and detection principle of fluorescent turn-on nanosensor MnIO@pep-FITC. (b) The detailed structure of MnIO@pep-FITC. The illustration is not drawn to scale.

The construction procedure and detection principle of as-proposed fluorescence turn-on nanosensor MnIO@pep-FITC was illustrated in Scheme 1. Using MnFe_2_(C_18_H_33_O_2_)_8_ as synthetic precursors, the OA capped MnIO NPs were firstly synthesized by thermal decomposition method with slight modification.^43–45^ The hydrophobic OA capped MnIO NPs were transferred into aqueous phase through ligand exchange of OA with DIB-PEG-COOH by formation of Mn(ii)/Fe(iii)-DIB complex.^42^ The MnIO@pep-FITC was constructed subsequently through the amidation reaction between the carboxy group of PEG on the DIB-PEG-COOH coated MnIO NP (MnIO@PEG) surface and amine group of FITC-pep. Herein, FITC was employed as the energy donor, while MnIO NP was used as the energy acceptor. The FITC dye was linked through a peptide substrate of trypsin with MnIO nanoparticles to establish a Förster resonance energy transfer (FRET) system for sensing the trypsin. The MnIO@pep-FITC exhibited weak fluorescence emission due to the strong interaction of MnIO NP with FITC. Due to the specific cleavage of C-terminal of arginine and lysine in the peptide by trypsin, the FITC dissociated from MnIO NP surface resulting in concomitant recovery of FITC fluorescence emission. The recovery of FITC fluorescence is proportional to the trypsin activity, enabling direct measurement/visualization of trypsin activity by fluorescence spectroscopy/imaging. Because of excellent magnetic property of MnIO NP, the sensitivity of MnIO@pep-FITC *in vivo* can be clearly improved through the external MF enhanced accumulation amount of MnIO@pep-FITC in tumor, creating the opportunity for sensitively visualizing small tumor.

**Fig. 1:**
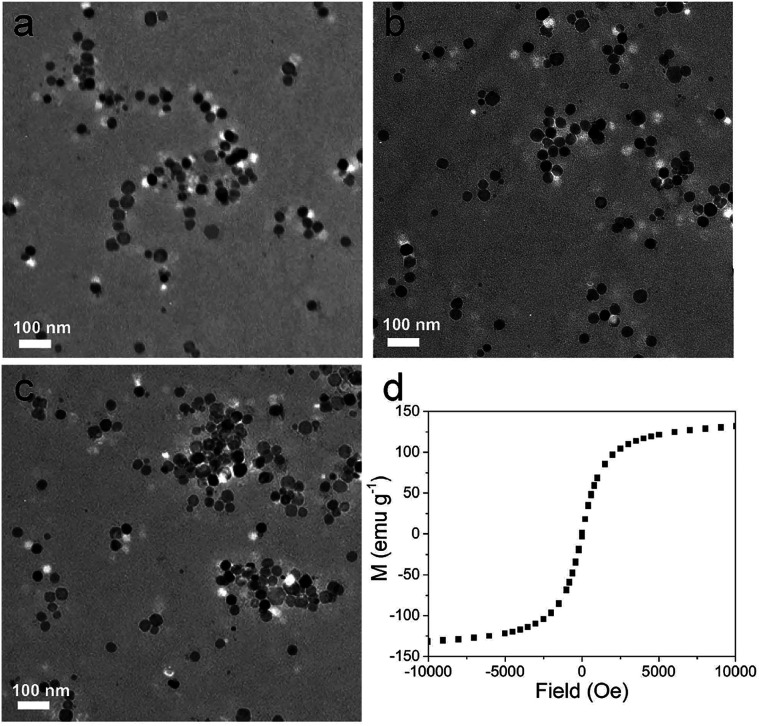
TEM micrographs of (a) MnIO, (b) MnIO@PEG, (c) MnIO@pep-FITC, respectively. (d) Magnetic hysteresis loop of MnIO@pep-FITC.

The size of as-synthesized OA capped MnIO NPs is 30.5 ± 2.9 nm in diameters (as shown in Fig. 1a). The X-ray diffraction (XRD) measurement indicates that the diffraction peaks of MnIO NPs is consistent with the manganese ferrite standard card (MnFe_2_O_4_, JCPDS 73-1964, as shown in Fig. S1†). The IR bands at 1443 cm^−1^ and 1653 cm^−1^ are attributed to C=C symmetric stretching vibration band of aromatic ring of the DIB group (as shown in Fig. S2†), suggesting that DIB-PEG-COOH is modified on the MnIO NPs surface. The IR band at 2088 cm^−1^ of FITC (vibration band of isothiocyanate) is observed in the FTIR spectrum of MnIO@pep-FITC (as shown in Fig. S2†), indicating that pep-FITC is successfully conjugated to MnIO-PEG. The size and morphology of MnIO NP exhibits negligible changes after modification with DIB-PEG-COOH and pep-FITC, respectively (as shown in Fig. 1b and c). The hydrodynamic sizes of MnIO-PEG and MnIO@pep-FITC are 53.77 ± 6.82 and 64.95 ± 4.87 (as shown in Table S1†), respectively, indicating that they have good monodispersity. Because of the out-layer of carboxyl group, the MnIO-PEG has relatively low zeta potential (−12.8 ± 1.01 mV, as shown in Table S1†). The zeta potential of MnIO@pep-FITC (−7.09 ± 0.56 mV) is higher than that of MnIO-PEG (as shown in Table S1†), also confirming the conjugation of pep-FITC with MnIO-PEG. The MnIO@pep-FITC shows relatively high saturation magnetization value (132 emu g^−1^ at 300 K, as shown in Fig. 1d), indicating that it has excellent superparamagnetic property. The fluorescence intensity of pep-FITC is decreased significantly after conjugation with MnIO-PEG (as shown in Fig. S3†). Based on fluorescence intensity results of original pep-FITC and supernatant, it can be calculated that there are about 429 pep-FITC molecules on one MnIO NP surface. The experimental result indicates that the fluorescence emission of FITC can be efficiently quenched by the MnIO NP. In addition, the fluorescence emission of FITC of MnIO@pep-FITC exhibits negligible changes after dispersed in different matrixes and/or stored in dark for 4 weeks (as shown in Fig. S4†), suggesting the good fluorescent and colloidal stability of MnIO@pep-FITC.

### 3.2 Sensing performance of MnIO@pep-FITC in buffer

**Fig. 2:**
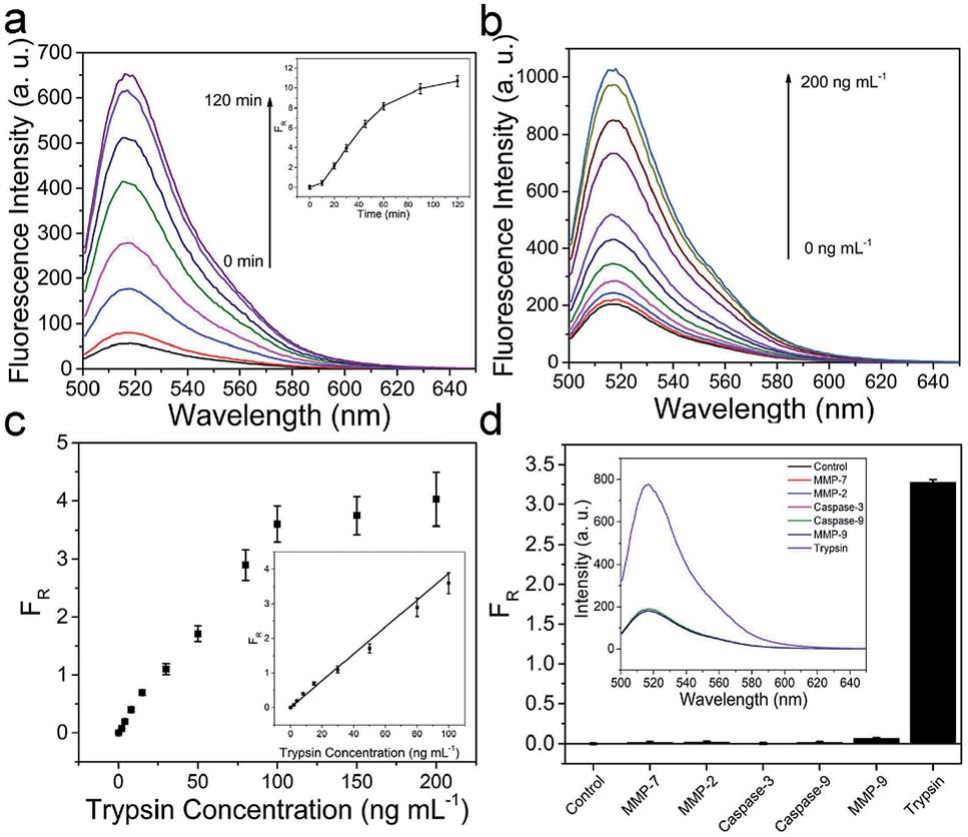
(a) Fluorescence spectra of 100 μg mL^−1^ MnIO@pep-FITC in the presence of 60 ng mL^−1^ trypsin at various incubation times. Inset is *F*_R_ of various incubation times. (b) Fluorescence spectra of MnIO@pep-FITC in the presence of various concentrations of trypsin following incubation for 90 min. (c) *F*_R_ of MnIO@pep-FITC as a function of trypsin concentration. (d) The selectivity of the MnIO@pep-FITC. The inset of (d) is the corresponding fluorescence spectra. The error bars mean standard deviations (*n* = 3).

The fluorescence intensity of FITC is gradually increased as the reaction time prolongs and reaches saturation at approximately 90 min, when the concentrations of trypsin and MnIO@pep-FITC are kept as constants (as shown in Fig. 2a). Taking together with the cytotoxicity of MnIO@pep-FITC, the trypsin is detected under following conditions, 90 min reaction time with 100 μg mL^−1^ MnIO@pep-FITC. The fluorescence intensity of FITC is increased proportionally with the concentration of trypsin increasing (as shown in Fig. 2b). The fluorescence recovery ratio (*F*_R_) has a good linear fit to the trypsin concentration within the range from 2 ng mL^−1^ to 100 ng mL^−1^ (as shown in the Fig. 2c). Here, *F*_R_ = Δ*F*/*F*_0_, Δ*F* = *F* − *F*_0_, *F*_0_ and *F* are the fluorescence intensities of FITC of MnIO@pep-FITC before and after trypsin digestion. The limit of detection is calculated to be 0.6 ng mL^−1^ or 25.2 pmol L^−1^ (3 times the standard deviation of *F*_R_ of blank solution), which is lower than those of literature reported.^46–48^ The performance of the method is better than or comparable to those of other methods (as shown in Table S2†). In order to evaluate its selectivity, MnIO@pep-FITC was exposed to five proteases including MMP-2, MMP-7, MMP-9, caspase-3 and caspase-9, which possibly coexist with trypsin in real samples (such as cells and tumor tissues). The *F*_R_ values of those interferences are much lower than that of trypsin (as shown in the Fig. 2d), suggesting that MnIO@pep-FITC has good selectivity.

### 3.3 Detection of intracellular trypsin activity

Prior to the detection of intracellular trypsin activity, the NCM460 cells (normal colonic epithelial cell) and HCT116 cells (CRC cell) were selected to evaluate the cytotoxicity of MnIO@pep-FITC by traditional MTT assay. The cell viabilities of NCM460 cells and HCT116 cells are still above 90% after incubated with 100 μg mL^−1^ MnIO@pep-FITC for 24 h (as shown in Fig. S5†). The result indicates that MnIO@pep-FITC has low cytotoxicity.

**Fig. 3:**
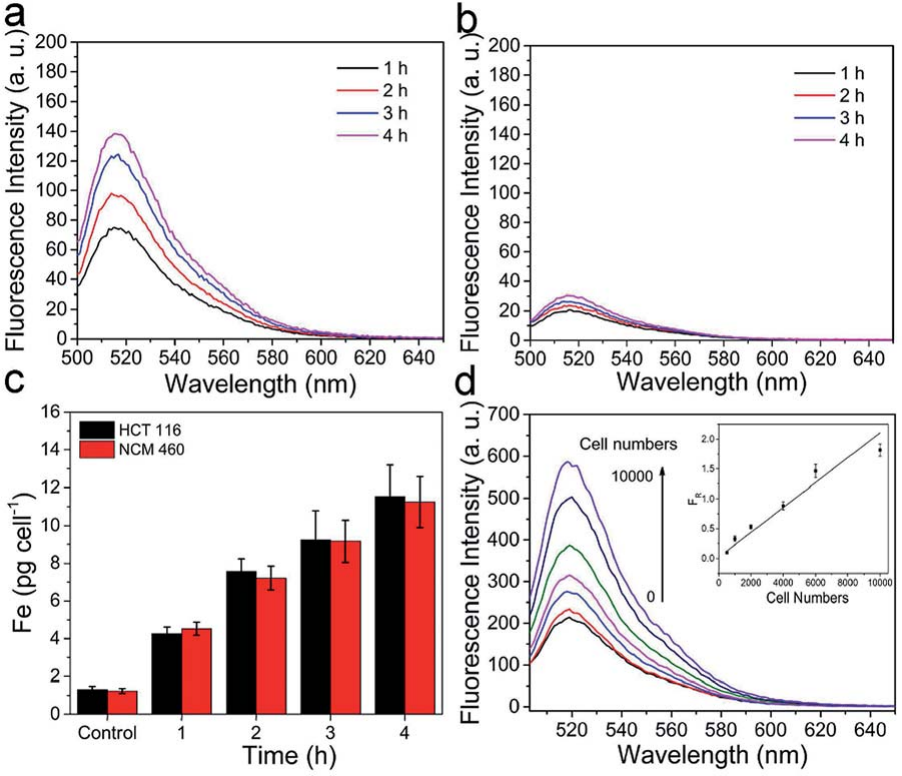
Fluorescence spectra of (a) HCT116 cells and (b) NCM460 cells cultured with 100 μg mL^−1^ MnIO@pep-FITC for various times (0–4 h), and the lysates of normal cultured HCT116 cells and NCM460 cells were used as backgrounds, respectively. (c) Corresponding ICP-MS results of HCT116 cells and NCM460 cells, and control groups are the results of normal cultured HCT116 cells and NCM460 cells. (d) The fluorescence spectra of 100 μg mL^−1^ MnIO@pep-FITC incubated with the lysates of various amounts of HCT116 cells. Inset is the corresponding calibration curve of *F*_R_*versus* cell numbers in 1 mL reaction solution. The error bars mean standard deviations (*n* = 3).

For demonstrating its sensing capability, MnIO@pep-FITC was employed for evaluating intracellular trypsin activities of NCM460 cells and HCT116 cells. As expected, the FITC fluorescence signals of cells were increased with increasing incubation time, when the cells were incubated with a certain concentration of MnIO@pep-FITC (as shown in Fig. 3a, b, S6 and S7†). Under the same experimental condition, the result of ICP-MS measurement demonstrates that there is no significant difference of internalization amounts between HCT116 cells and NCM460 cells (as shown in Fig. 3c), while the fluorescence intensity of HCT116 cells is much higher than that of NCM460 cells. The results demonstrate that HCT116 cells express higher level of trypsin than that of NCM460 cells. In addition, the fluorescence intensity of mixtures is gradually increased with increasing cell numbers when MnIO@pep-FITC were incubated with HCT116 cell lysates. And the *F*_R_ value shows a linear correlation with the cell lysate within the range of 5 × 10^2^ to 1 × 10^4^ cells per mL with a detection limit of 359 cells per mL. The result also demonstrates that MnIO@pep-FITC has high sensitivity for detection of intracellular trypsin activity.

**Fig. 4:**
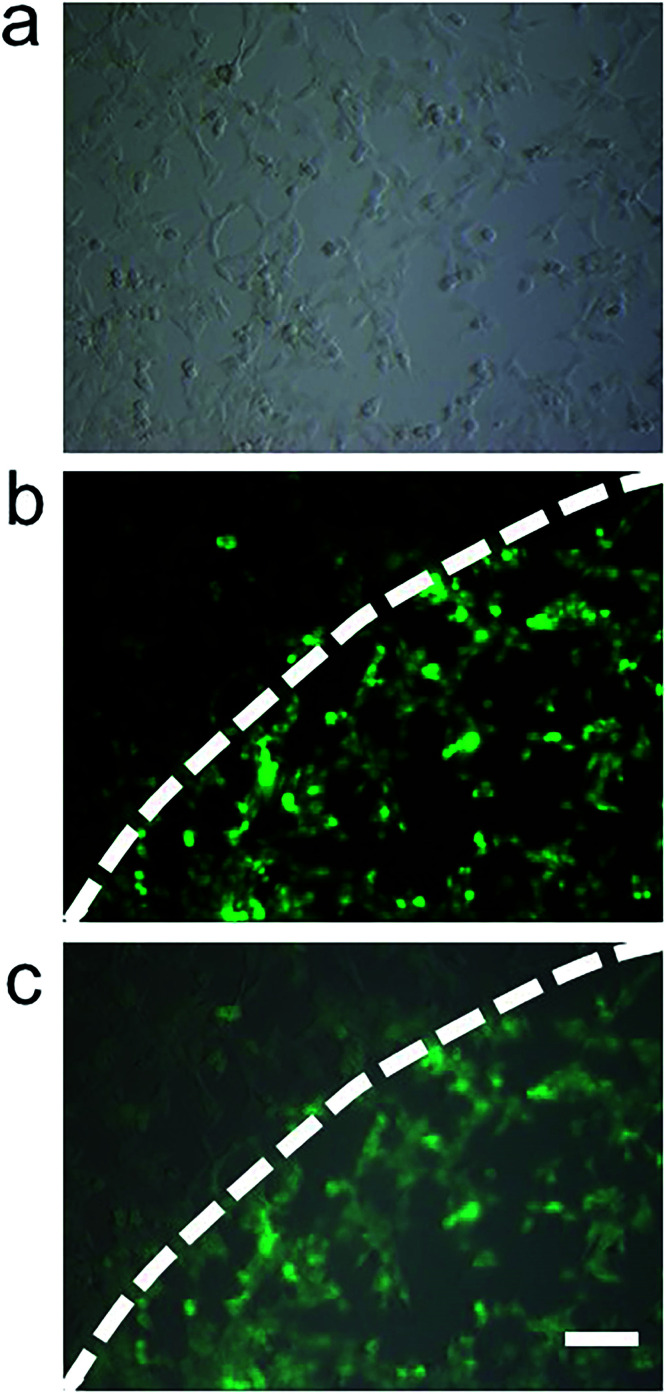
Fluorescence micrographs of HCT116 cells co-cultured with 100 μg mL^−1^ MnIO@pep-FITC in the MF for 4 h, (a) bright field mode, (b) FITC mode and (c) merging image. The scale bar is 100 μm.

Furthermore, there is an apparent local accumulation of MnIO@pep-FITC guided by the MF (*i.e.*, only the cells localized close to the magnet show strong fluorescence signal (as shown in Fig. 4)). All of the experimental results suggest that MnIO@pep-FITC could be used to sensitively visualize tumor through *in situ* monitoring of trypsin activity in cancer cells with the aid of external MF.

### 3.4 Detection of trypsin activity *in vivo*

Before detection of trypsin activity *in vivo*, the toxicity of MnIO@pep-FITC was evaluated by blood test and histology analysis. There is little difference in blood examinations between control group and MnIO@pep-FITC treated group (as shown in Table S3†). No hemolytic phenomenon is observed by hemolysis test (as shown in Fig. S8†). The histology analysis suggests that there is neither noticeable tissue damage nor inflammation of MnIO@pep-FITC on major organs (as shown in Fig. S9†). These results demonstrate that MnIO@pep-FITC has good biocompatibility.

**Fig. 5:**
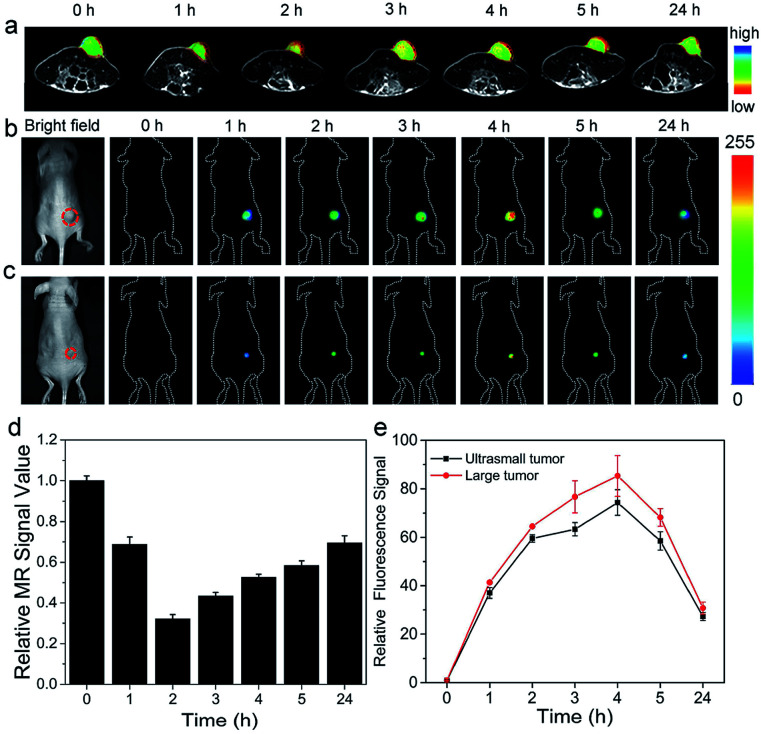
(a) *In vivo* MR images of HCT116 tumor-bearing nude mice injected intravenously with MnIO@pep-FITC (0 h means pre-injection). (b and c) *In vivo* fluorescence images of HCT116 tumor-bearing nude mice with intravenous injection of MnIO@pep-FITC (0 h means pre-injection, (b) large tumor (126 mm^3^ in volume) and (c) ultrasmall tumor (*ca.* 4.9 mm^3^ in volume)). (d) Relative MR signal values of tumor after intravenous injection of MnIO@pep-FITC at different time intervals. (e) Quantification of fluorescence intensities at the tumor sites of (b) and (c) after intravenous injection. The error bars mean standard deviations (*n* = 3).

For demonstrating its sensing capability *in vivo*, MnIO@pep-FITC were injected into in the HCT116 tumor-bearing BALB/c nude mice through the tail veins with external MF assistance, respectively. The *in vivo* T_2_-weighted MR and fluorescence imaging were performed after intravenous injection of MnIO@pep-FITC. Generally, the tumor site displays a clearly signal enhancement on both of the MR images and FITC fluorescence images at 1 h post-injection of MnIO@pep-FITC (as shown in Fig. 5). The MR signal enhancement is gradually decreased after reaching maximum MR signal enhancement at 2 h post-injection (as shown in Fig. 5a and d). The fluorescence intensity of FITC continuously increases until it reaches the maximum value at 4 h post-injection before decaying (as shown in Fig. 5b and e). The delay of maximum fluorescence intensity of FITC to maximum MR signal can be understood by the cleavage kinetics of trypsin (as shown in Fig. 2a) because the fluorescence intensity of FITC is mainly governed by the trypsin activity. The results suggest that MnIO@pep-FITC can be employed to *in situ* monitor trypsin activity of tumor by *in vivo* fluorescence imaging. Encouraging by its strong trypsin detection capacity, MnIO@pep-FITC was injected into nude mice-bearing ultrasmall HCT116 tumor xenografts (4.9 mm^3^ in volume) through the tail veins. Strong fluorescence signal enhancement of tumor site was successfully observed at 4 h post-injection (as shown in Fig. 5c and e). The result further demonstrates that MnIO@pep-FITC could be used as an efficient tool to diagnose tumor through sensitive measurement of trypsin activity.

The *in vivo* MR images of mice organs were recorded for evaluating the *in vivo* clearance pathway of the MnIO@pep-FITC. During the whole experiment process, the MR signals of liver exhibit significant variation, while MR signals of kidneys show negligible change (as shown in the Fig. S10 and S11†). Only a small amount of MnIO@pep-FITC accumulated in the kidney and were excreted within 24 hours. The MR signal of liver decreased by 42% at 5 h post-injection. And after 24 hours, the degree of MR signal reduction returned to 31%. The result demonstrates that MnIO@pep-FITC are metabolized by the liver.

## 4. Conclusions

In summary, a fluorescence turn-on nanosensor, MnIO@pep-FITC has been developed for highly sensitive and selective determination of trypsin activity both *in vitro* and *in vivo*. The results indicate that the MnIO@pep-FITC has relatively low detection limits for both of pure trypsin and intracellular trypsin integrated with a reasonable dynamic range. In particular, the MnIO@pep-FITC enables to noninvasively visualize BALB/c nude mouse-bearing ultrasmall (*ca.* 4.9 mm^3^ in volume) trypsin-positive subcutaneous tumor by *in vivo* fluorescence imaging with the aid of an external MF. Combination with its excellent biocompatibility and long-term stability, the MnIO@pep-FITC shows great promise for screening trypsin-positive tumors at early stage.

## Conflicts of interest

There are no conflicts to declare.

## Supplementary Material

RA-011-D0RA08171J-s001
