# Supplementary material for: Peptide modified manganese-doped iron oxide nanoparticles as a sensitive fluorescence nanosensor for non-invasive detection of trypsin activity in vitro and in vivo
Source: RSC Adv. 2021 Jan 11;11(4):2213–20. doi: 10.1039/d0ra08171j (PMC8693661; doi:10.1039/d0ra08171j)
Supplement: RA-011-D0RA08171J-s001 [file RA-011-D0RA08171J-s001.pdf]

Electronic Supplementary Material (ESI) for RSC Advances.
This journal is © The Royal Society of Chemistry 2020

Electronic supplementary information for
Peptide Modified Manganese-Doped Iron Oxide Nanoparticle as a Sensitive Fluorescence Nanosensor for Non-invasive Detection of Trypsin Activity in Vitro and in Vivo

Yu Fu [a,b], Lin Liu [a,c], Xiaodong Li [b], Hongda Chen[c,*], Zhenxin Wang [c], Wensheng Yang [a], Hua Zhang[c,*] and Huimao Zhang[b,*]

[a]*College of Chemistry, Jilin University, Changchun 130021, P. R. China*

[b]*Department of Radiology, The First Hospital of Jilin University, Changchun 130021, P. R. China*

[c]*State Key Laboratory of Electroanalytical Chemistry, Changchun Institute of Applied Chemistry, Chinese Academy of Sciences, Changchun 130022, P. R. China*

Tel.: +86-431-85262757

Fax: +86-431-85262243

E-mail address: chenhongda@ciac.ac.cn (Hongda Chen), zhanghua@ciac.ac.cn (Hua Zhang), huimao@jlu.edu.cn (Huimao Zhang)

Contents

1. Additional Experimental Section

2. Additional Figures S1-S11

3. Additional Tables S1-S3

4. Additional Reference

1. Additional Experimental Section

1.1 Instruments

Transmission electron microscope (TEM) micrographs and high-magnification transmission electron microscope (HRTEM) micrographs were obtained on a FEI Tecnai G2 S-Twin TEM (FEI Co., USA) with a field emission gun operating at 200 kV. The X-ray diffraction (XRD) patterns of as-prepared samples were carried out on a D8 advance diffractometer (Bruker Co., Germany) using Cu Kα (0.15406 nm) radiation. Fourier transform infrared (FTIR) spectra were captured with a Bruker Vertex 70 spectrometer. The X-ray photoelectron spectroscopy (XPS) measurements were conducted with a VG ESCALAB MKII spectrometer (VG Scientific Ltd., UK). All dynamic light scattering (DLS) and Zeta potential distribution measurements were carried out on the Malvern Zetasizer Nano ZS (Malvern Instruments Ltd., UK). The element analysis experiments were performed by an ELAN 9000/DRC ICP-MS system (Perkin Elmer, USA). The fluorescence emission spectra were recorded on a QE65 Pro fiber optic spectrometer (Ocean Optics Ltd., USA). The fluorescence micrographs were performed with a reconstructive Ti-S fluorescent microscope (Nikon Co., Japan). The in vivo fluorescence imaging was observed by the Davinch Invivo HR imaging system (Davinch K, Korea).

1.2 Synthesis of OA capped $MnFe_2O_4$ nanoparticles

The metal-oleate precursor, $MnFe_2(C_{18}H_{33}O_2)_8$ was prepared by reaction of sodium oleate with the correspondent metal chloride salts. Briefly, 4 mmol $FeCl_3$, 2 mmol $MnCl_2$, and 16 mmol sodium oleate were dissolved in the mixture of 10 mL ethanol, 10

mL Milli-Q water and 20 mL hexane. The reaction mixture was heated to reflux at 60 °C for 4 h. After removal of the aqueous phase, the organic phase was heated to 70 °C for another 2 h. Then, the mixture was heated to 110 ºC under vacuum for 2 h to remove residual solvent. Finally, a viscous metal-oleate product was obtained. The $MnFe_2O_4$ nanoparticles (MnIO NPs) were synthesized by the literature reported strategy [1]. 6 mmol $MnFe_2(C_{18}H_{33}O_2)_8$ and 3 mmol OA were dissolved in 12 mL ODE and evacuated for 30 min. The solution was heated to 200 ºC under argon flow, following heated to 330 ºC at a rate of 1 °C min^{-1}, and maintained at 330 ºC for 1 h. The products were precipitated by adding isopropanol (45 mL), then collected by centrifugation (6000 rpm, 10 min) and redispersed in hexane for further use.

1.3 Cell viability measurements

The HCT116 and NCM460 cells (1×10^4 cells per well) were cultured with 100 μL fresh culture medium containing 10% FBS and 100 U mL^{-1} penicillin-streptomycin in 96-well microtiter plate under a humidified 5% CO_2 at 37 °C for 24 h, respectively. Specially, McCoy's 5A culture medium was used for culturing HCT116 cells, while RPMI 1640 culture medium was used for culturing NCM460 cells. After washed with 100 μL PBS (3 times), the cells were treated with various concentrations (0, 6.25, 12.5, 25, 50 and 100 μg mL^{-1}) of MnIO@pep-FITC and incubated for another 24 h, respectively. Cell viability was determined by conventional MTT assay. The relative viabilities of MnIO@pep-FITC stained cells and untreated cells (control sample) were calculated via measuring the absorbance at 490 nm by a microplate reader.

2. Additional Figures S1-S9

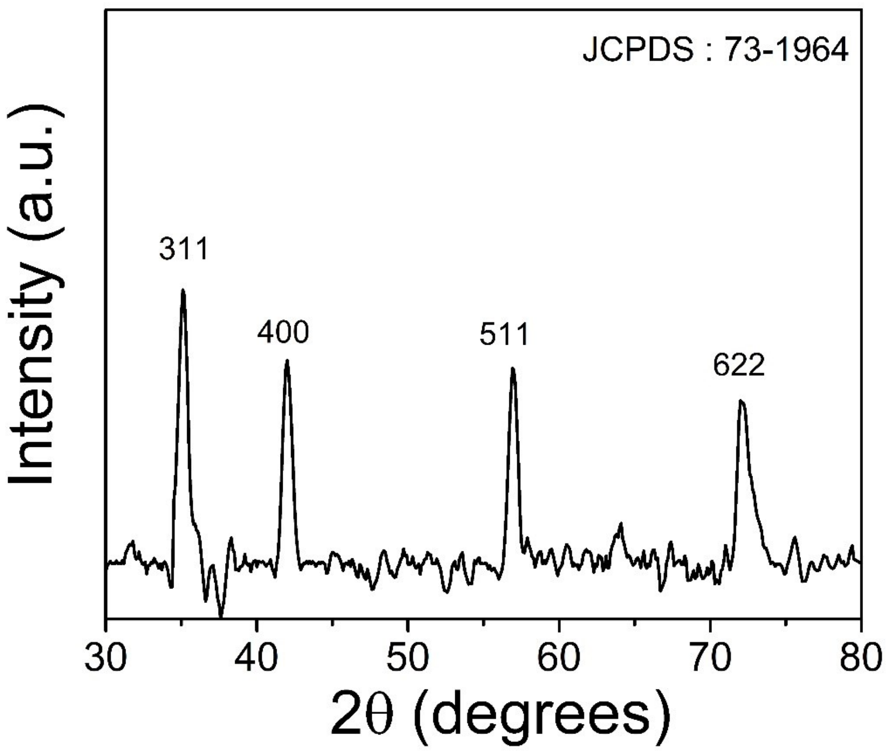

Fig. S1 The X-ray diffraction (XRD) patterns of MnIO NPs.

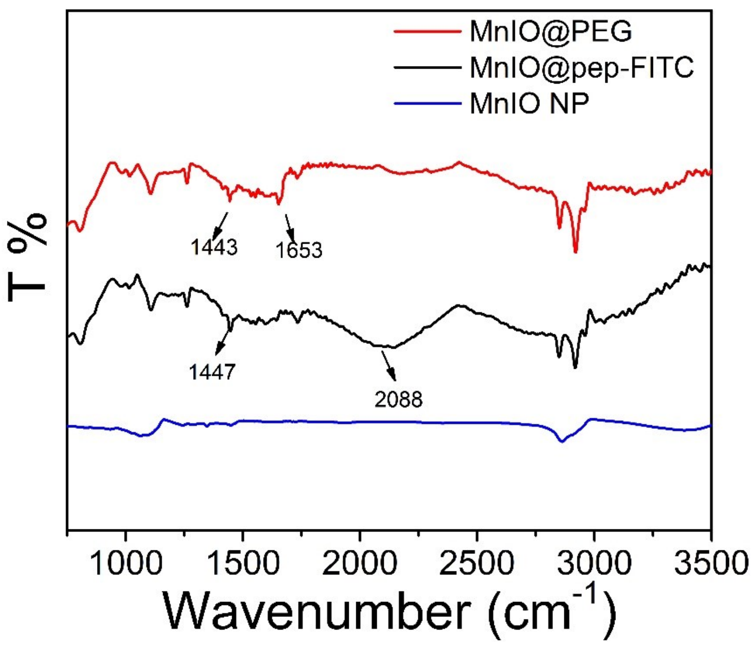

Fig. S2 FTIR spectra of MnIO NPs, MnIO@PEG and MnIO@pep-FITC, respectively.

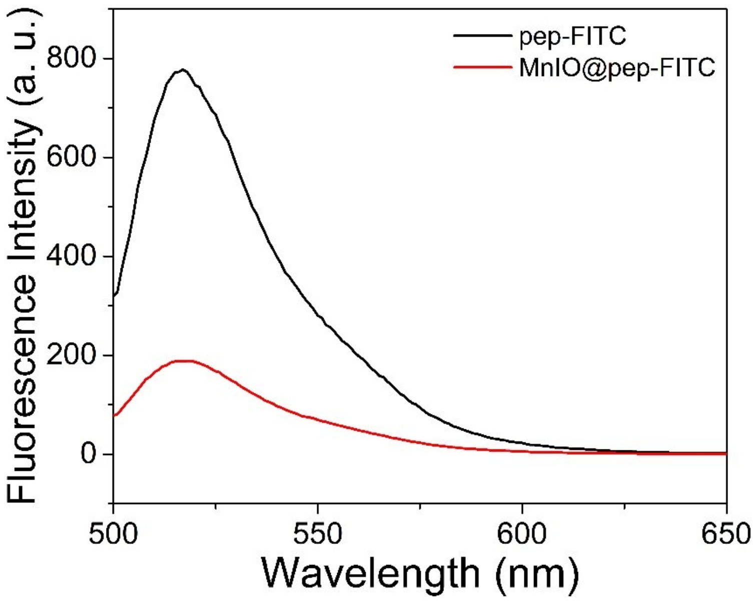

Fig. S3 The fluorescence spectra of pep-FITC and MnIO@pep-FITC.

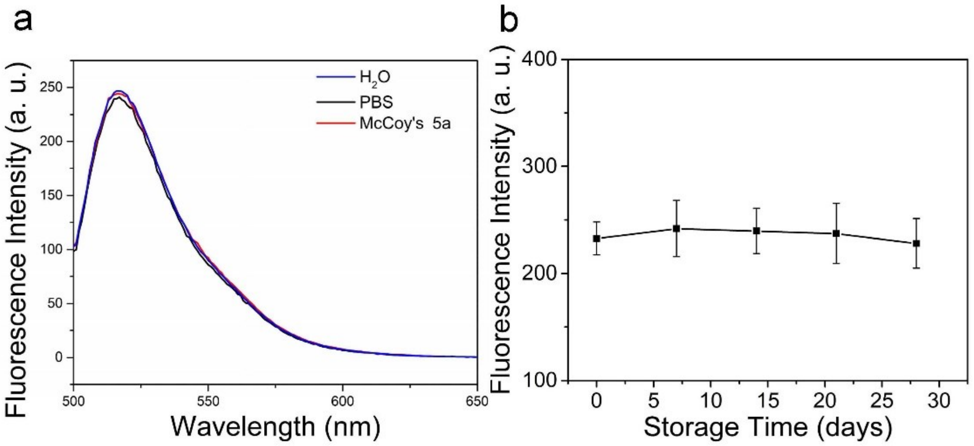

Fig. S4 a) The fluorescence spectra of 100 μg mL^{-1} MnIO@pep-FITC dispersed in H_2O, PBS and McCoy's 5A culture medium. b) The maximum fluorescence intensity of MnIO@pep-FITC dispersed in PBS as a function of storage days.

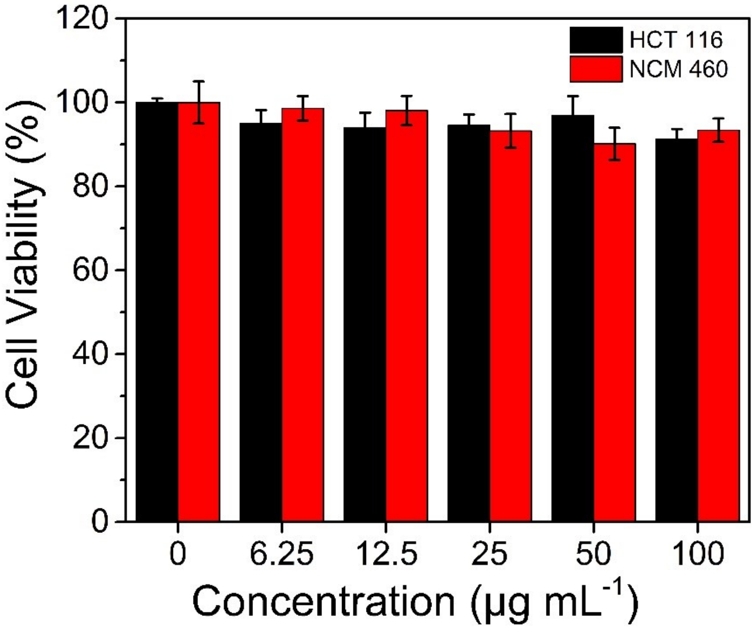

Fig. S5 Cell viabilities of HCT116 cells and NCM460 cells as a function of the MnIO@pep-FITC concentration.

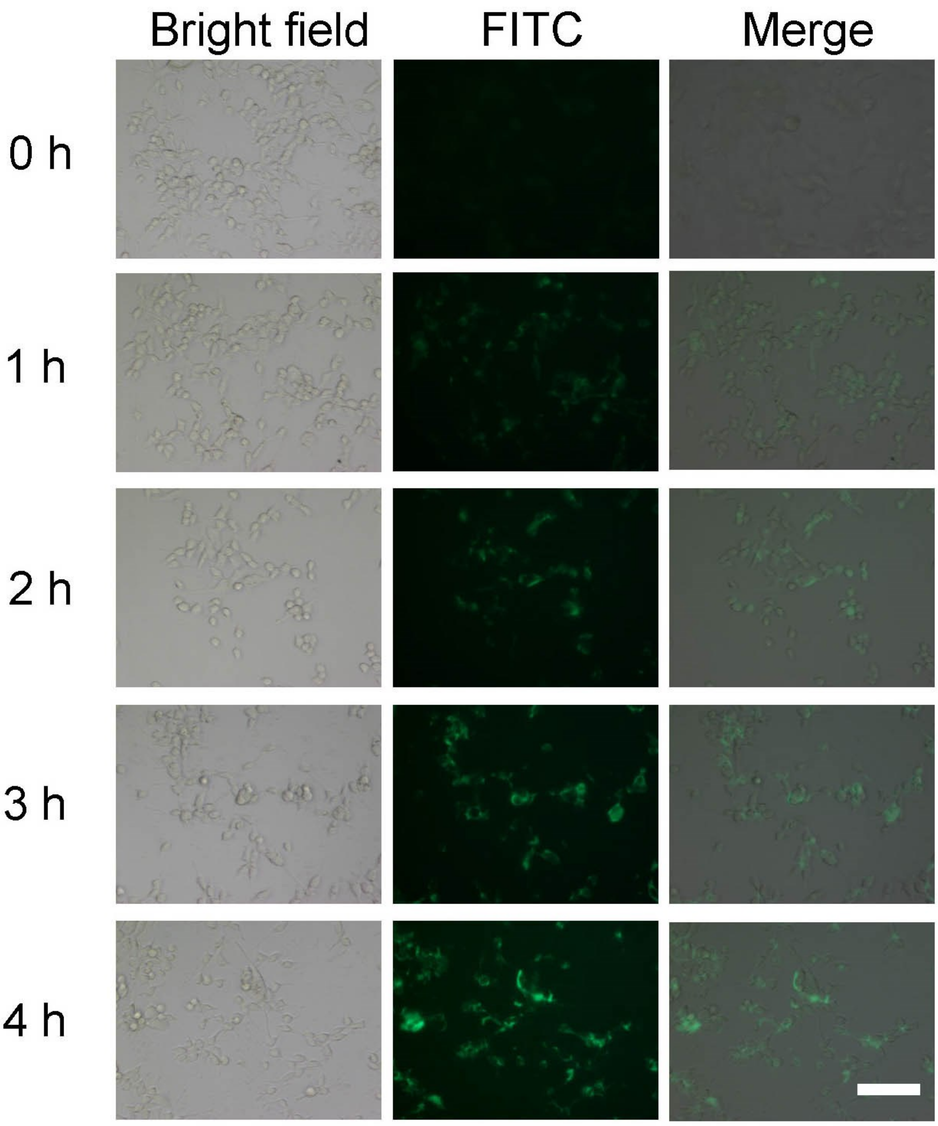

Fig. S6 Fluorescence micrographs of HCT116 cells co-cultured with 100 μg mL^{-1} MnIO@pep-FITC for various times (0-4 h). The scale bar: 100 μm.

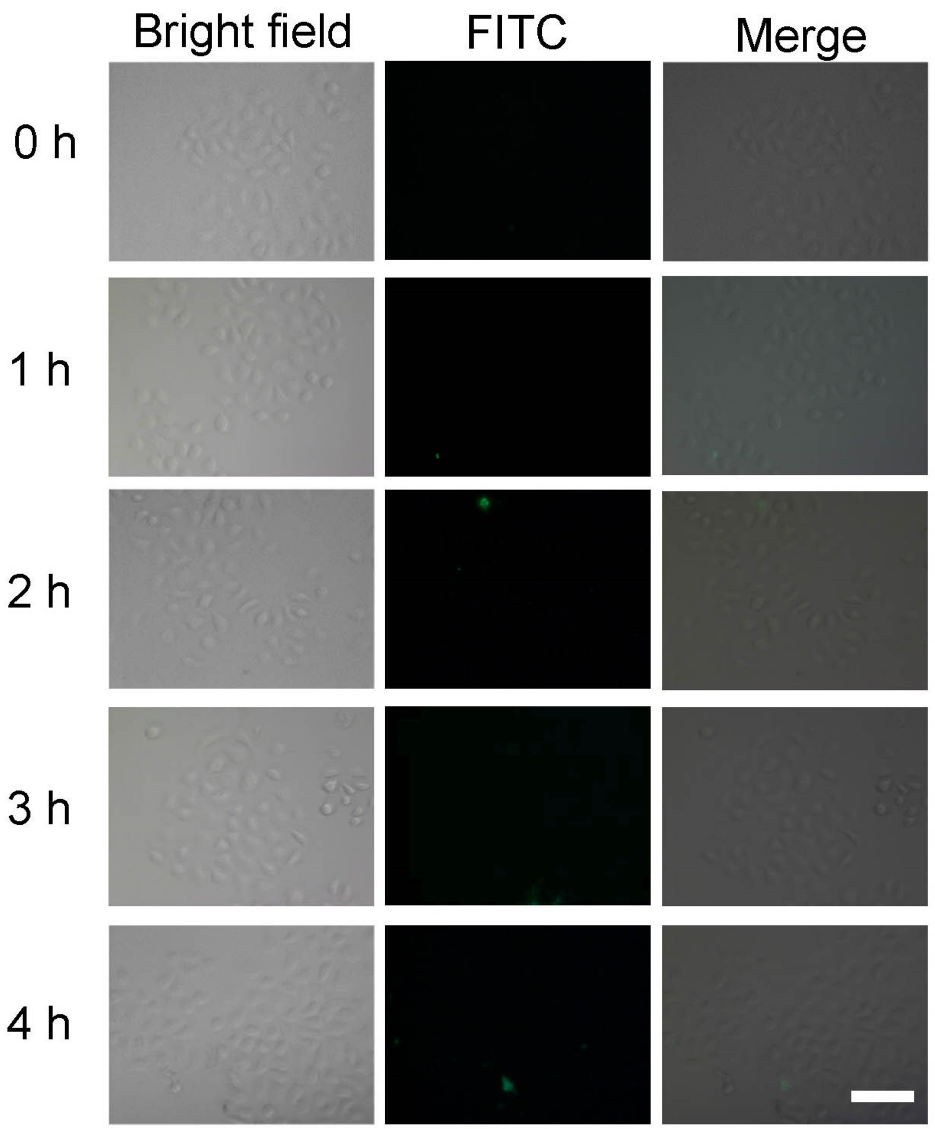

Fig. S7 Fluorescence micrographs of NCM460 cells co-cultured with 100 μg mL^{-1} MnIO@pep-FITC for various times (0-4 h). The scale bar: 100 μm.

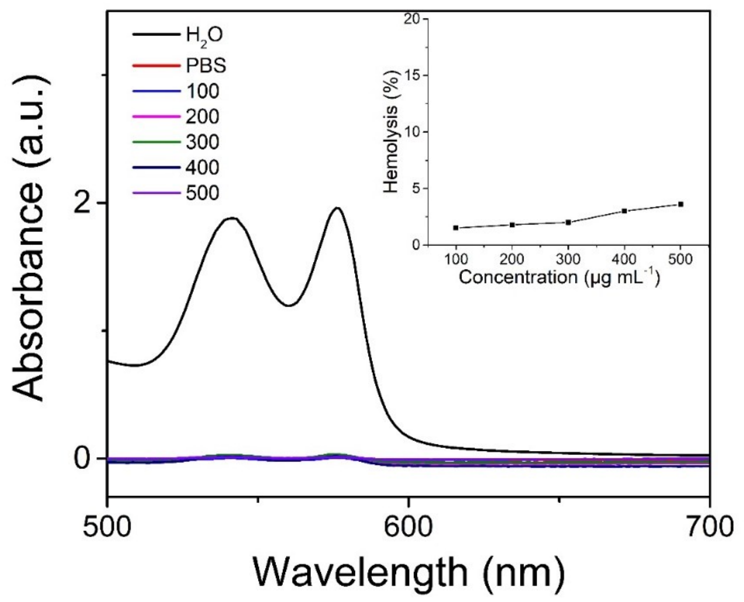

Fig. S8 Hemolysis assay of MnIO@pep-FITC. PBS is used as negative control while H_2O is used as positive control, respectively. Inset illustrates the hemolysis percentage with different concentrations of MnIO@pep-FITC (100, 200, 300, 400 and 500 μg mL^{-1}).

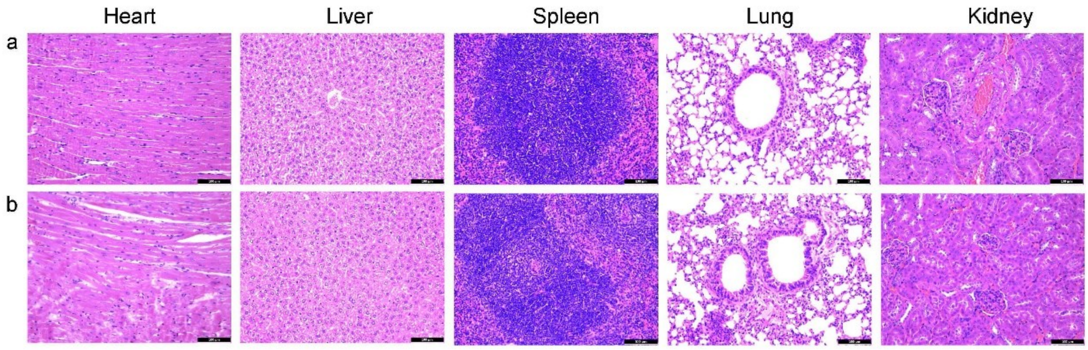

Fig. S9 Histological changes of healthy mice at 30-day post-injection with a) saline and b) MnIO@pep-FITC, respectively.

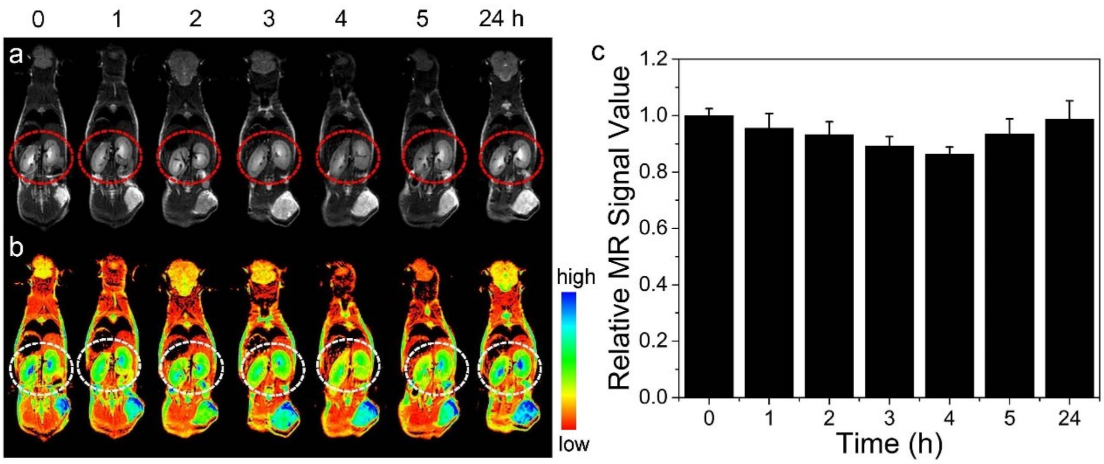

Fig. S10 In vivo MR images of kidneys (ellipses) after the treatment of MnIO@pep-FITC (0 h means pre-injection), (a) MR images, (b) pseudo-color of MR images and (c) corresponding relative MR signal values, respectively.

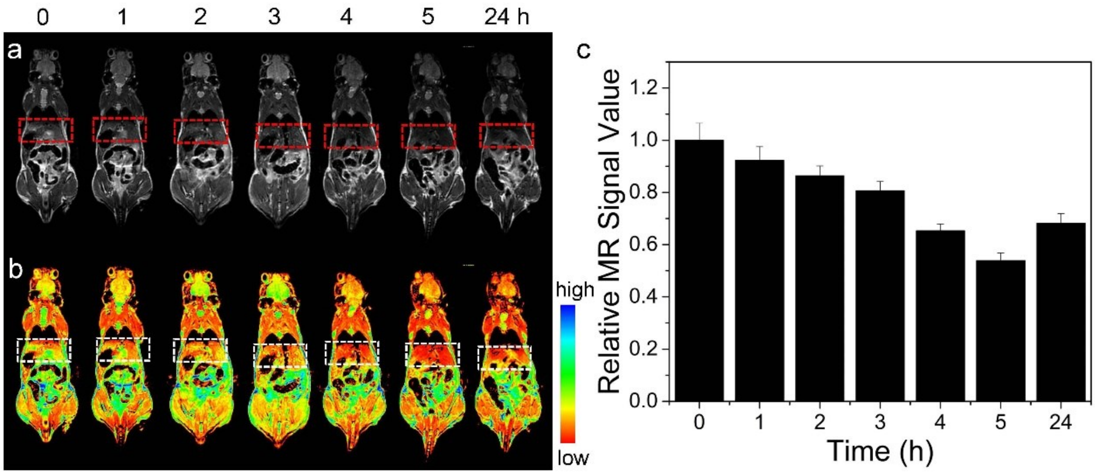

Fig. S11 In vivo MR images of liver (rectangles) after the treatment of MnIO@pep-FITC (0 h means pre-injection), (a) MR images, (b) pseudo-color of MR images and (c) corresponding relative MR signal values, respectively.

3. Additional Tables S1-S3

Table S1 Zeta potentials and hydrodynamic sizes of nanoparticles in water.

Sample	Zeta potential (mV)	Hydrodynamic size (nm)
MnIO@PEG	-12.8 ± 1.01	53.77 ± 6.82
MnIO@pep-FITC	-7.09 ± 0.56	64.95 ± 4.87

Table S2 Analytical performance of different methods toward trypsin detection.

Method	Detection limit ($ng\ mL^{-1}$)	Linear response range ($ng\ mL^{-1}$)	Ref
UCNP-peptide-AuNP	4.15	12–208	2
Gold nanoclusters	4	10-2000	3
Silver triangular nanoplates	1.8	5–80	4
silver nanoparticles	2	2.5-200	5
carbon dots and gold nanoparticles	0.84	2.5-80	6
MnIO@pep-FITC	0.6	2-100	This work

Table S3 Results of blood biochemical assays.

Parament	Units	Control	Treatment
WBC	10^9 L^{-1}	1.38	1.66
LYMPH	%	75.78	82.74
RBC	10^{12} L^{-1}	11.34	10.50
HGB	g L^{-1}	172	166
HCT	%	59.10	57.32
MCV	fL	48.52	48.49
MCH	pg	15.30	14.90
MCHC	g L^{-1}	308.47	314.09
PLT	10^9 L^{-1}	157	168

4. Additional Reference

(1) Chen, R.; Christiansen, M. G.; Anikeeva, P. Maximizing Hysteretic Losses in Magnetic Ferrite Nanoparticles via Model-Driven Synthesis and Materials Optimization. ACS Nano 2013, 7, 8990-9000.

(2) Wu, M.; Wang, X.; Wang, K.; Guo, Z. An ultrasensitive fluorescent nanosensor for trypsin based on upconversion nanoparticles. Talanta 2017, 174, 797-802.

(3) Zhao, D.; Chen, C.; Zhao, J.; Sun, J.; Yang, X. Label-free fluorescence turn-on strategy for trypsin activity based on thiolate-protected gold nanoclusters with bovine serum albumin as the substrate. Sensors and Actuators B-Chemical 2017, 247, 392-399.

(4) Chen, H.; Fang, A.; Zhang, Y.; Yao, S. Silver triangular nanoplates as an high efficiently FRET donor-acceptor of upconversion nanoparticles for ultrasensitive "Turn on-off" protamine and trypsin sensor. Talanta 2017, 174, 148-155.

(5) Miao, P.; Liu, T.; Li, X.; Ning, L.; Yin, J.; Han, K. Highly sensitive, label-free colorimetric assay of trypsin using silver nanoparticles. Biosensors & Bioelectronics 2013, 49, 20-24.

(6) Xu, S.; Zhang, F.; Xu, L.; Liu, X.; Ma, P.; Sun, Y.; Wang, X.; Song, D. A fluorescence resonance energy transfer biosensor based on carbon dots and gold nanoparticles for the detection of trypsin. Sensors and Actuators B-Chemical 2018, 273, 1015-1021.
